# Characterization of physiochemical and nutrient profiles in canola feedstocks and co-products from bio-oil processing: impacted by source origin

**DOI:** 10.5713/ab.22.0410

**Published:** 2023-02-26

**Authors:** Alessandra M. R. C. B. de Oliveira, Peiqiang Yu

**Affiliations:** 1 Department of Animal and Poultry Science, College of Agriculture and Bioresources, University of Saskatchewan, Saskatoon, SK, S7N 5A8, Canada

**Keywords:** Canola Bio-oil Processing, Feedstock and Co-products, Nutritional Value, Physiochemical Profiles, Precision Feeding, Source Origin

## Abstract

**Objective:**

The objective of this study was to characterize physiochemical and nutrient profiles of feedstock and co-products from canola bio-oil processing that were impacted by source origin. The feedstocks and co-products (mash, pellet) were randomly collected from five different bio-oil processing plants with five different batches of samples in each bio-processing plant in Canada (CA) and China (CH).

**Methods:**

The detailed chemical composition, energy profile, total digestible nutrient (TDN), protein and carbohydrate subfractions, and their degradation and digestion (CNCPS6.5) were determined.

**Results:**

The results showed that TDN_1x_ was similar in meals between CA and CH. CH meals and feedstock had higher, truly digestible crude protein (tdCP) and neutral detergent fiber (tdNDF) than CA while CA had higher truly digestible non-fiber carbohydrate (tdNFC). The metabolizable energy (ME_3x_), net energy (NE_Lp3x_, NE_m3x_, and NE_g3x_) were similar in meals between CA and CH. No differences were observed in energy profile of seeds between CA and CH. The protein and carbohydrate subfractions of seeds within CH were similar. The results also showed that pelleting of meals affected protein sub-fractionation of CA meals, except rapidly degradable fractions (PB1), rumen degradable (RDPB1) and undegrdable PB1 (RUPB1), and intestinal digestible PB1 (DIGPB1). Canola meals were different in the soluble (PA2) and slowly degradable fractions (PB2) between CA and CH. The carbohydrate fractions of intermediately degradable fraction (CB2), slowly degradable fraction (CB3), and undegradable fraction (CC) were different among CH meals. CH presented higher soluble carbohydrate (CA4) and lower CB2, and CC than CA meals.

**Conclusion:**

The results indicated that although the seeds were similar within and between CA and CH, either oil-extraction process or meal pelleting seemed to have generated significantly different aspects in physiochemical and nutrient profiles in the meals. Nutritionists and producers need to regularly check nutritional value of meal mash and pellets for precision feeding.

## INTRODUCTION

Canola has been produced in Western Canada since 1974, when it was developed as a low erucic acid and low glucosinolate rapeseed, to supply for the high demand of cooking oil [1]. When canola oil is extracted, it generates a co-product low in fat and rich in protein. This co-product, canola meal, is mainly used in dairy rations because its amino acid profile is ideal for milk synthesis [2].

Due to the high production of canola and the high global demand, besides being ex tensively used in Canada, it is also exported to many countries. China is one of the main markets for Canadian canola seeds and its product and co-product [3].

Different crops and seed processing methods can alter the composition of the nutrients [4] and the protein profile of canola meals. Meaning that canola meals should not be assumed equal before prior to proper testing.

Canola meal is a co-product that contains outstanding rumen degradable (RDP) and undegradable protein (RUP) profiles that stimulates both microbial growth and milk synthesis [5]. White et al [6] defended the prediction of RUP because of its importance for dairy rations, as RUP content can impact both the microbial protein synthesis and the amino acid profile that will be available for absorption in the small intestine of the animal.

The aim of this study was to characterize the physiochemical composition and nutrient profiles of canola seeds and meals from five different large oil-seed crushing plants in Canada and five different large oil-seed crushing plants in China, using standard wet laboratory analyses, and the NRC [7,8] and CNCPS 6.5 models.

## MATERIALS AND METHODS

The University of Saskatchewan Animal Care Committee approved the animal trial under the Animal Use Protocol No. 19910012 and animals were cared for and handled in accordance with the Canadian Council of Animal Care (CCAC) regulations [9]. Authors confirm that EU and Canadian standards for the protection of animals and/or feed legislation have been met.

### Sampling

The samples of feedstocks and co-products from bio-oil processing were arranged and collected from Canada and China by the Canola Council of Canada (CCC). The samples were provided by each company’s quality control laboratory and are to be considered representative of the reality of those crushers.

Samples were collected from five crusher companies op erating in four provinces in China. These companies only crushed seeds imported from Canada. Samples of seeds and meals were collected from different batches from each crusher, stored and transported to the University of Saskatchewan in Canada for further analyses.

Samples of seeds and meals were also collected from five crushers in Canada. However, three of the five Canadian crushers samples of meals were pelleted and two were mash, unlike China’s meals that were all mash. Samples were collected from different batches from each crusher, stored and transported to the University of Saskatchewan for future analyses.

All samples of seeds were ground using a blade coffee grinder, model BCG111OB manufactured by KitchenAid, USA. The samples of meals that were pelleted at a low temperature (ca 70°C) were ground using a 1mm screen on the grinding mill, Ultra Centrifugal Mill ZM200 manufactured by Retsch, Germany.

### Chemical analysis

The chemical analysis of the samples followed the analytical procedures described on the Official Methods of Analysis 21st Edition [10] for dry matter (DM), ash, crude protein (CP), ether extract (EE), neutral detergent fiber (NDF), acid detergent fiber (ADF), lignin, hemicellulose, cellulose, non-fiber carbohydrate (NFC), non-structural carbohydrate. For neutral detergent insoluble CP (NDICP) and acid detergent insoluble CP (ADICP), the procedures by Licitra et al [11] were followed. To determine the soluble CP (SCP) content of the samples, the methodology by Roe et al [12] was applied.

### Total digestible nutrient and energy profile

The digestible nutrient profiles (tdFA, tdCP, tdNDF, tdNFC, and TDN) and energy values (DE, ME, NE_g_, NE_m_, NE_L_) of the feedstocks and co-products from bio-oil processing were determined based on the chemical profile, according to the National Research Council (NRC): Nutrient Requirements for Dairy Cattle [7] and Nutrient Requirements for Beef Cattle [8].

### Protein and carbohydrate fraction partitioning with CNCPS 6.5 System

The Cornell Net Carbohydrate and Protein System (CNCPS) partitions carbohydrates and proteins into fractions based on rates of passage and digestion. Carbohydrates were fractionated into volatile fatty acids (CA1), lactic acid (CA2), other organic acids (CA3), water soluble carbohydrate (CA4), soluble fiber (CB2), digestible fiber (CB3), and indigestible fiber (CC). The protein fractions correspond to ammonia (PA1), soluble true protein (PA2), insoluble or moderately digestible true protein (PB1), fiber-bound or slowly digestible protein (PB2), and unavailable or indigestible protein (PC) [13–15]. Following the fractionation of proteins and carbohydrates, the ruminal degradation and intestinal digestion were also studied.

### Statistical analysis

To better accommodate the variations and prevent statistical errors, the statistical design of this study was a randomized complete block design, where country and company were fixed effects and batch was a random effect. The procedure MIXED was used on SAS 9.4 (SAS Institute, Cary, NC, USA).


$$y=\mu +\tau_{i}+\beta_{j}+{ɛ}_{ijk}$$

Where, *μ* = overall mean; *τ_i_* = fixed effect; *β_j_* = random effect; *ε_ij_* = error; β_j_ ~ NIID (Normally, Identically, and Independently distributed); ε_ijk_ ~ NIID (Normally, Identically, and Independently distributed). The samples from different batches from each processing plant were used as experiment units. Significance was declared at p<0.05. The Tukey method was used for the multiple comparison test.

## RESULTS AND DISCUSSION

### Chemical profiles of feedstocks and co-products: comparison among bio-oil processing plants and between two countries

The chemical profile of canola meals is presented in Tables 1 and 2. In this study, canola meals DM averaged higher on the samples collected in Canada than on the samples from China (p<0.001). Crude protein was lower for the Canadian samples (p = 0.003). Ether extract was not different between Canadian and Chinese samples (p = 0.118). Acid detergent fiber was also similar between countries (p = 0.408).

According to the Canadian Oilseed Processors Associa tion (COPA) [16], a maximum of 12% moisture and 12% of crude fiber, and a minimum of 36% of protein and 2% of fat (solvent extracted, measured in % by mass) are the standard specifications for canola meal. The 2020 Canola Annual report (CCC, n.d.) [17] complied data from 7 years with samples from 13 different Canadian plants and found as average chemical composition that canola meals had 42% CP, 3.2% EE, 18.6% ADF (on a DM basis), and 12% moisture.

Paula et al [18] reported CP as 41.8%DM, NDF as 28.9% DM, and ADF as 18.6%. On a review, Paula et al [19] reported canola meal with 91.4% of DM, 39.8% DM of CP, 19.4% DM of ADF, 28.5% DM of NDF, and 4.56% DM of EE. Mustafa et al [20] reported the profile of canola meal as 42% DM of CP, 24% DM of NDF, and 19% DM of ADF. While Broderick et al [21] reported using canola meal with 89.6% of DM, 40.6% DM of CP, 3.0% DM of EE, 29.9% DM of NDF, 18.2% DM of ADF, 26.2% CP of NDICP, and 6.2% CP of ADICP.

Based on these results, the canola meal samples analyzed for this project were in accordance with these values previously reported, except for the EE which was lower than reported by Paula et al [18,19] and CCC [17] and expected by COPA [16]. Our EE values for canola meals averaged 0.79% DM for the samples from Canada and 0.47% DM for the ones from China; however, the samples from plants 3 and 4 from Canada that were pelleted presented EE of 1.46 and 1.06% DM respectively, which can be associated with the coating of the pellets with oil, but this higher EE was not observed on the pellets from plant 5 (0.63% DM).

Soluble crude protein and neutral detergent indigestible crude protein (NDICP or NDIP) were different between Canada and China. While China presented higher CP (p = 0.003) and SCP (p<0.001), Canada presented higher NDICP (p<0.001). Acid detergent insoluble crude protein (ADICP) was not significantly different (p = 0.075, Table 1).

Mustafa et al [20] stated that the NDICP of regular canola meal was 105 g/kg CP which is lower when compared to meals from Canada that averaged 19.34% CP, but close to the samples from plants A and C from China (11.07% and 10.83% CP), however, still lower than China’s average of 13.52% CP. They also reported ADICP as 45 g/kg CP was lower than this project’s meals (Canada [5.53% CP] and China [5.80% CP]).

According to Newkirk [4], different cultivars, canola growth environments and harvest, and the processes the seeds and meals go through can all affect the final nutrient profile of the meal. Since five different companies were sampled in the production of different batches of meals both in Canada and in China, it is safe to assume that these results are representative of the companies and their quality is steady through different batches, and small variations are expected due to the variability of crop conditions, cultivars, and harvest.

The chemical profile of the canola seeds studied on this project is displayed in Tables 3 and 4. The DM of seeds from Canadian plants was higher than those from Chinese plants (p = 0.008). Crude protein content was similar (p = 0.100, Canada vs China). Soluble CP was higher for China plants (p = 0.002). And NDICP was higher for Canada plants (p<0.001). Neutral detergent fiber, ADF and cellulose were higher for Canada plants (p = 0.004, p = 0.003, and p<0.001, respectively), while ADL was higher for the China plants (p = 0.017).

Park et al [22] studied samples of canola seeds, canola meals from solvent extraction and canola meals from expellers. For canola seeds, they reported DM of 94.9%, ash of 3.04% DM, CP of 24.8% DM, NDF of 19.4% DM, and ADF of 15.5% DM. Averaging Canada and China together and comparing to these results, our seeds had higher moisture content (92.7%), higher ash (3.8% DM), lower CP (22.3% DM), lower NDF (16.4% DM), and lower ADF (12.1% DM).

Canola seeds used by Tramontini [23] were composed of 23.51% DM of CP, 37.34% DM of EE, and 26.52% DM of NDF. Tramontini’s seeds were higher in CP and NDF contents (ours were 22.3% DM and 16.4% DM, respectively), and lower in EE (ours averaged 43.3% DM).

The Canadian Grain Commission (CGC) [24] summa rized the canola seed production of 2020 and observed an oil content of 44.1% DM and CP of 20.8%. On their report from the 2015 [25] production, they observed seeds with 44.2% DM EE and 20.7% DM of CP. These results give us a basis to safely assume that Canada produces canola with a high and stable along the years.

Burbulis and Kott [26] investigated the variation in color and oil content influenced by the environmental temperature on black-seeded spring rapeseed varieties *Brassica napus* L. ‘Bolero’ (owned by Raps GbR) and ‘Star’ (owned by Dansk Planteforaedling/DLF) and 11 lines originated from their crossing. They found that temperatures higher than 28°C during the day, resulted in offspring with lighter seeds (more yellow) and temperatures lower than 20°C resulted in darker seeds (more brown or black). They also observed differences in oil content on the seeds from different environments. The oil content of the darker seeds (colder climate) ranged from 31.2% to 51.6% DM, and lighter seeds (warmer climate) ranged from 31.4% to 49.4% DM. On average, lighter seeds presented lower oil content.

Tramontini [23] likely used canola seeds from a different climate, since her study was conducted in Brazil, a tropical country with higher temperatures, as Burbulis and Kott [26] study suggests, the higher temperatures in that country could have influenced the seeds she used, explaining the lower EE content. The seeds analyzed on our project, however, were in accordance with the standard quality of the Canadian canola seeds.

The higher cellulose content on the Canada plants (p < 0.001) could have been the cause for higher contents of NDF (p = 0.004), ADF (p = 0.003), and NDICP (p<0.001) on the samples from that country.

### Total digestible nutrients and digestible (DE), metabolizable (ME), and net energy (NE) values of feedstocks and co-products: comparison among bio-oil processing plants and between two countries

The energetic profile of canola meals and pellets are represented in Tables 5 and 6. Total digestible NDF (tdNDF), total digestible CP (tdCP), and total digestible nutrients (TDN_1x_) were different among Canadian plants (p<0.001, p = 0.001, and p = 0.001, respectively). The contrast indicated that the meals pelleted (Plants 3, 4, and 5) resulted in higher tdNDF and TDN_1x_ (p<0.001, and p = 0.002) than the mash. When pelleting, it is common practice to add back to the process fines collected during the screening step and that might have contributed to a lightly higher tdNDF in this study. Also, as a final step of pelleting, there is the spraying of oil to increase the durability of the pellet, which might have been the cause for a slightly higher TDN_1x_ on Plant 3. tdNDF and tdCP were also variable among the meals from Chinese plants (p<0.001 and p = 0.002). When analyzing the overall meals from Canada and China, it was observed that tdNDF, tdNFC, and tdCP were different (p<0.001, p = 0.006, and p<0.001), of these, Canada had higher tdNFC, while China presented higher tdNDF and tdCP.

Metabolizable energy at three times maintenance (ME _3x_), net energy for lactation (NE_Lp3x_), maintenance (NE_M3x_), and gain (NE_g3x_) were all observed to be different among the meals from the Canadian plants (p<0.001, for all of them). Differences between mash and pelleted meals were also observed of these parameters (p<0.05) with the Plant 3 showing the higher results. While these differences were present on the Canadian samples, no differences were observed on the Chinese samples. Moreover, the overall comparison of canola meals from Canada and China showed that they are similar.

Damiran et al [27] reported using canola meal with 42.6% of CP, 4.2% of fat, 71.5% of TDN, 2.0 Mcal/g of NE_m_, and 1.3 Mcal/g of NE_g_. While this study’s Canadian canola meal averaged 41.9% of CP, 0.79% of EE, 65.6% of TDN, 1.8 Mcal/g of NE_m_, and 1.2 Mcal/g of NE_g_. Theodoridou and Yu [28] analyzed canola meals from yellow and brown canola seeds and showed some differences in their energy profiles. Therefore, the higher TDN (71.5%) on Damiran et al [27] might be explained by that canola meal being from a yellow seeded cultivar or as a consequence of the higher fat and protein content of that meal, since the TDN value is based on the values of digestible carbohydrates, protein and fat of a feedstuff [29].

The energy profile of canola seeds is displayed in Tables 7 and 8. As expected, the seeds presented less variations. No differences were observed on the digestible nutrients profile from Canadian plants. Only the tdCP of canola seeds from the Chinese companies were different in this study (p = 0.006). This might be due to the varieties difference. The overall comparison of the energetic parameters of canola seeds from Canada and China only the tdNDF from Canadian plants were higher (p = 0.023), while all the other parameters were similar. Similar values were observed for ME_3x_, NE_Lp3x_, NE_m3x_, and NE_g3x_ on all samples collected in Canada and in China.

### Protein and carbohydrate subfractions and degradable and digestible content of each fraction in rumen phase and intestinal phase using newly updated CNCPS System 6.5 for feedstocks and co-products

Table 9 presents the protein fractions of canola meals and pellets based on the CNCPS 6.5 System. For the Canadian canola meals and pellets, it was observed that for the soluble fraction of protein (PA2) of the canola meals mash and pellets, the Plant 4 presented the highest amount while Plant 1 presented the lowest. For the moderately degradable fraction (PB1), Plant 3 had the highest value, and Plant 2 had the lowest among the companies. On the slowly degradable protein fraction (PB2), while Plant 2 resulted in the highest value for the fraction, Plant 4 had the lowest. Plant 3 presented the lowest amount of unavailable protein (PC), whereas Plant 2 the highest. The contrast analysis also showed differences between the mash and pelleted meals for soluble, slowly degradable, and unavailable fractions of protein (PA2, p = 0.008; PB2, p = 0.003; PC, p<0.001). Possibly, the conditioning step of the pelleting process, that uses high temperatures, influenced the protein structures of the meals, and consequently increased their availability for degradation. All fractions were different among the Chinese plants (PA2, p<0.001; PB1, p = 0.021; PB2, p<0.001; PC, p<0.001). However, the comparison between the Canadian and Chinese protein fractions of the meals showed that only PA2 (p<0.001) and PB2 (p<0.001) were different, with China having higher soluble and lower slowly degradable fractions than Canada.

The ruminal degradable and undegradable, and intestinal digestible fractions profile of the Canadian and Chinese canola meals and pellets are shown in Table 10–16. In accordance with the results from Table 9, Table 15 shows that Plant 4 presented higher RDPA2 (p = 0.038) and RUPA2 (p = 0.036), and lower RDPB2 (p = 0.002), RUPB2 (p = 0.002), and DIGPB2 (p = 0.002); and Plant 2 had lower RDPB1 (p = 0.003), RUPB1 (p = 0.003), and DIGPB1 (p = 0.003). Because of the higher amounts of soluble true protein, Plant 4 presented lower amounts of intestinal digestible feed protein (p<0.001). There were no differences between the meals and pellets on the amounts of RDPB1, RUPB1, and DIGPB1 fractions.

The Chinese meals presented variations in the ruminal degradability of PA2, PB2, peptides, and total ruminal degradable protein fractions (p<0.001, for all); on the ruminal undegradable PA2, PB2, PC, and total rumen undegradable protein fractions (p<0.001, p<0.001, p<0.001, and p = 0.006, respectively); and on the intestinal digestible PB2 and feed protein (DIGFP) fractions (p<0.001 and p = 0.039).

While the rumen degradable fractions of PA2, PEP, and total RDP, and the rumen undegradable fraction of PA2 were higher in the Chinese meals (p<0.001), the rumen degradable PB2, rumen undegradable PB2, and intestinal digestible PB2 and FP fractions were higher for the Canadian meals. Higher availability of protein in the rumen (degradable fractions) guarantees enough amino acid supply for the rumen microbiota, however higher availability of protein for intestinal digestion and absorption (intestinal digestible fractions) means that a higher variability of amino acids will be available for the animal to use for muscle deposition and milk production.

The protein fractions of the canola seeds analyzed in this study are represented in Table 10. The Canadian seeds presented some variation on the contents of PB2, PC, and TP fractions (p<0.001, for all). The Canadian Plant 2 had the highest content of PB2, while Plant 5 presented the lowest. Plant 4 showed higher content of PC and lower content of TP. The opposite was observed on Plant 3 that showed the lowest PC and the highest TP. All the seeds from the five different Chinese companies were similar for all protein fractions presented. Only the slowly degradable fraction (PB2) was different between Canada and China (p<0.001), where Canadian seeds presented higher amounts of this fraction.

The rumen and intestinal fractions are presented in Table 16, where we see a similar ruminal degradation and intestinal digestion profile. The RDPB2, RUPB2, RUPC, and DIGPB2 are different among Canadian plants (all p<0.001). No difference is observed among the seeds from the various Chinese plants, and RDPB2, RUPB2, and DIGPB2 are higher in the seeds from Canada (p<0.001).

Li et al [30] analyzing co-products from canola bio-ener gy processing found PA2, 26.8% CP; PB1, 63.6% CP; PB2, 7.0% CP; and PC, 2.6% CP. And predicted RDPA2, 7.7% DM; RDPB1, 13.9% DM; RDPB2, 0.7% DM. Total RDP, 22.3% DM; RUPA2, 2.6% DM; RUPB1, 10.5% DM; RUPB2, 2.0% DM; RUPC, 1.0% DM; and total RUP, 16.1% DM. The values for PA2, RDPA2, RDPB1, and total RDP are higher than the ones found for canola meals on this study. And their contents of PB2, PC, RDPB2, RUPB1, RUPB2, RUPC, and total RUP are lower than ours. However, we had similar results for PB1 and RUPA2.

The carbohydrate fractions of canola meals and pellets are given in Table 11. Canadian canola meals different among the five plants for digestible (CB3) (p = 0.002) and indigestible fiber (CC) (p<0.001). Plant 4 showed the lowest amount of digestible fiber (CB3) and the highest of indigestible fiber (CC). Plant 5 displayed the highest content of CB3 and Plant 3 the lowest amount of CC. Only the CC fraction showed a difference between the mash and pelleted meals (p<0.001). The Chinese meals presented variability among companies on the CB2, CB3, and CC fractions (p = 0.012, p = 0.013, and p = 0.010, respectively). The Chinese plant B showed higher quantities of CB2 and CC than the other companies. And Plant D had higher amount of CB3. Besides these differences, Canadian and Chinese meals were only different on the content of CA4, CB2, and CC (p = 0.040, p = 0.010, and p<0.001).

The predicted rumen degradable and undegradable and intestinal digestible carbohydrate fractions are revealed in Table 13. The rumen degradable CB3 (RDCB3), rumen undegradable CB3 (RUCB3), and intestinal digestible CB3 (DIBCB3) fractions were found to be the highest on Plant 5, and the lowest on Plant 2 (p<0.001, for all three). Total rumen undegradable carbohydrate (total RUC) was the highest on Plant 2 and the lowest on Plant 3 (p<0.001). The intestinal digestible feed carbohydrate (DIGFC) was the highest on Plant 5 and the lowest on Plant 3. The contrast analysis showed that pelleting influenced the RUCC and total RUC fractions of the canola meals on this study (p<0.001, for both). The rumen degradable and undegradable CA4, CB2, and CB3 fractions were variable among the Chinese plants (p<0.05). Plant E presented the highest values for RDCA4 and RUCA4 (p = 0.018, p = 0.018, respectively). Plant B showed the highest amounts of RDCB2 (p = 0.014) and RUCB2 (p = 0.014). Plant D resulted in the highest contents of RDCB3, RUCB3, and DIGCB3 (p = 0.007, for all). The rumen degradable, undegradable, and intestinal digestible CB2, the RUCC, and total RUC fractions of canola meals were higher in the Canadian companies (p = 0.009, p = 0.008, p = 0.008, p<0.001, and p = 0.009, respectively).

Table 12 presents the carbohydrate fractions of canola seeds from Canadian and Chinese companies. Only the CB2 and CC fractions seemed to be different among companies (p = 0.002 and p<0.001, respectively), where Plant 3 showed the lowest values for both. All the samples analyzed from the five Chinese samples were similar. Only the amounts of water-soluble CHO (CA4) and digestible fiber (CB3) differed between countries (p = 0.022 and p = 0.006).

Table 14 shows the predicted amounts of rumen degradable and undegradable and intestinal digestible carbohydrate fractions of canola seeds. This table shows that while Plant 5 exhibited the highest values of rumen degradable, undegradable, and intestinal digestible CB2, and total RDC, the Plant 3 exhibited the lowest values for those variables (p = 0.003, p = 0.003, p = 0.003, and p = 0.020, respectively). Apart from DIGFC (p = 0.043), all other variables analyzed on the Chinese canola seeds were similar. And excluding the CB3 fractions (RDCB3, RUCB3, and DIGCB3; p = 0.006 for these three), all other fractions are similar between the canola seeds analyzed from Canadian and Chinese companies.

Huang [31] reported a study on different temperatures and conditioning time during the pelleting of canola meals and showed that neither the carbohydrate fractions nor the predicted rumen degradable and undegradable carbohydrate fractions were affected by the different treatments. This finding is in accordance with our results because only the indigestible fiber fractions (CC, RUCC, and total RUC) expressed a difference between mash and pellets (p<0.001, for the three fractions).

### Summary and conclusion

#### Summary

The chemical profile of canola meals from Canada and China presented significant differences on DM, ash, CP, SCP, and NDICP. Whereas the chemical profile of canola seeds from Canada and China presented differences on DM, SCP, NDICP, NDF, AF, ADL, and cellulose. Because variations can be caused by crop environment, cultivar, and processing, these differences do not seem relevant.

The pelleting of canola meals by the Canadian companies seemed to have influenced tdNDF and TDN_1x_. On the other hand, the meals from China were not pelleted and differences were observed on tdNDF and tdCP. On the overall comparison of the mash meals, China presented higher tdNDF, and tdCP, and lower tdNFC than Canada.

The energy values of canola seeds were very similar among companies on Canada and China except for tdCP on the Chinese samples that showed some variations among plants. Between countries, only tdNDF was higher in Canada. No differences were observed on the energy values (NE_Lp3x_, NE_m3x_, and NE_g3x_) of canola seeds from China or Canada.

The protein fractions of the canola meals from Canada and China were similar, except for PA2 and PB2, where PA2 was higher in China and PB2 in Canada. The content of PB2 was also higher for the Canadian seeds. RDPA2, RUPA2, RDPEP, and total RDP were higher on the Chinese meals, whereas RDPB2, RUPB2, DIGPB2, and DIGPF were higher on the Canadian meals. While the Chinese seeds presented higher amounts of RDPB2, RUPB2, and DIGPB2.

The Chinese meals and seeds showed higher content of water-soluble carbohydrates (CA4). Canadian meals presented higher soluble (CB2) and indigestible (CC) fiber contents, and consequently higher RDCB2, RUCB2, RUCC, and DIGCB2 than the ones from China. The meals from Canada were also higher in RUCC and Total RUC. While the rumen degradable, undegradable and intestinal digestible fractions of CB3 were higher in Canada, all the other variables were similar between the two countries.

#### Conclusion

From this study, we can conclude that the canola seeds used by the companies from both countries are not different in chemical and nutrient profiles, and that the canola meals can present some variations depending on the processing (oil processing and meal pelleting) it went through in the crushing plants. The chemical composition, protein and carbohydrate fractions, TDN value, energy value and nutrient supply for lactation, growth and maintenance differed between the countries. This indicated that the oil processing and extract methods and meal processing either mash or pelleting significantly affected nutritional value. For practice purpose, nutritionists and producers need to regularly check nutritional value of meal and pellets for precision feeding.

## Figures and Tables

**Table 1:** Chemical composition profile of co-products from different oil processing plants (canola meal and pellet): comparison among bio-oil processing plants and between Canada and China

Items	Basic chemical profile				Protein profile						
	
	DM (%)	Ash (% DM)	EE (% DM)	FA (% DM)	CP (% DM)	SCP (% DM)	SCP (% CP)	NDICP (% DM)	NDICP (% CP)	ADICP (% DM)	ADICP (% CP)
	------------------------------------------------------------------------- CA processing plants -------------------------------------------------------------------------										
Plant 1 (M)	90.28	7.60^b^	0.68	0.47	42.62^a^	7.08^b^	16.63^b^	7.65^ab^	17.95^ab^	2.47^a^	5.80^a^
Plant 2 (M)	89.49	8.24^a^	0.79	0.44	40.94^b^	7.05^ab^	17.20^ab^	8.70^a^	21.30^a^	2.45^ab^	5.98^a^
Plant 3 (P)	83.13	8.25^a^	1.46	1.11	41.64^b^	7.92^ab^	19.01^ab^	6.03^bc^	14.51^b^	2.02^c^	4.87^b^
Plant 4 (P)	89.83	7.43^b^	1.06	0.74	41.70^b^	8.57^a^	20.55^a^	6.03^c^	14.48^b^	2.37^ab^	5.69^a^
Plant 5 (P)	89.25	7.28^b^	0.63	0.28	41.84^ab^	7.46^ab^	17.82^ab^	7.83^a^	18.73^ab^	2.30^b^	5.49^a^
SEM	0.497	0.094	0.718	0.552	0.222	0.468	1.070	0.443	1.123	0.091	0.225
p-value	0.281	<0.001	0.606	0.604	0.001	0.037	0.017	0.001	0.001	<0.001	<0.001
	------------------------------------------------------------------------------- Meal vs Pellet -------------------------------------------------------------------------------										
Contrast p-value	0.188	0.004	0.472	0.477	0.766	0.014	0.008	0.001	0.001	<0.001	<0.001
	------------------------------------------------------------------------- CH processing plants -------------------------------------------------------------------------										
Plant A (M)	88.21	7.05	0.82	0.38	42.71^bc^	9.53^b^	22.33^b^	4.74^b^	11.07^b^	2.14^bc^	5.00^bc^
Plant B (M)	88.54	7.09	0.41	0.02	43.31^abc^	9.45^b^	21.77^bc^	7.05^a^	16.27^a^	2.85^a^	6.60^a^
Plant C (M)	88.52	7.42	0.74	0.35	43.25^ab^	11.01^a^	25.46^a^	4.69^b^	10.83^b^	2.05^c^	4.75^c^
Plant D (M)	88.89	6.72	0.50	0.10	43.87^a^	10.19^ab^	23.24^ab^	6.30^a^	14.35^a^	2.08^c^	4.74^c^
Plant E (M)	88.56	7.27	0.43	0.03	42.17^c^	8.16^c^	19.36^c^	6.60^a^	15.62^a^	2.42^b^	5.74^b^
SEM	0.311	0.202	0.415	0.236	0.321	0.341	0.862	0.473	1.046	0.090	0.209
p-value	0.615	0.112	0.554	0.599	0.003	<0.001	<0.001	<0.001	<0.001	<0.001	<0.001
	------------------------------------------------------------------------------------- Overall -------------------------------------------------------------------------------------										
CA Plants	89.96	7.89	0.79	0.46	41.87	7.15	17.11	8.07	19.34	2.45	5.86
CH Plants	88.55	7.12	0.47	0.16	43.04	9.71	22.51	5.83	13.52	2.29	5.33
SEM	0.285	0.143	0.397	0.211	0.305	0.365	0.851	0.48	1.125	0.104	0.245
p-value	<0.001	<0.001	0.118	0.125	0.003	<0.001	<0.001	<0.001	<0.001	0.192	0.075

For each plant, sample size n = 5.

DM, dry matter; EE, ether extract (crude fat); FA, fatty acid; CP, crude protein; SCP, soluble crude protein; NDICP, neutral detergent-insoluble crude protein; ADICP, acid detergent-insoluble crude protein; CA, Canada; CH, China; SEM, standard error of the mean.

a–c Means within a column without a common superscript letter differ (p<0.05).

**Table 2:** Chemical composition profile of co-products from different oil processing plants (canola meal and pellet): comparison among bio-oil processing plants and between Canada and China

Items	Carbohydrate profile												

	CHO (% DM)	Sugar (% DM)	Sugar (% NFC)	NDF (% DM)	ADF (% DM)	ADF (% NDF)	ADL (% DM)	ADL (% NDF)	HEM (% DM)	Cell (% DM)	NFC (% DM)	NFC (% CHO)	NSC (% DM)
	------------------------------------------------------------------------------------- CA processing plants ------------------------------------------------------------------------------------------------------------												
Plant 1 (M)	48.98	8.72	33.80	30.73^bc^	20.03^c^	65.10^bc^	9.65^bc^	31.62^ab^	10.75^a^	10.36^b^	25.95	53.17^ab^	14.20
Plant 2 (M)	50.14	7.97	31.06	33.26^a^	21.90^a^	66.31^bc^	10.59^a^	31.80^a^	11.24^a^	11.37^a^	25.64	51.06^b^	14.72
Plant 3 (P)	48.78	9.10	33.98	27.89^d^	19.36^c^	69.70^ab^	7.92^d^	28.28^b^	8.45^b^	11.48^a^	26.99	55.22^a^	14.87
Plant 4 (P)	49.81	9.58	36.70	29.92^cd^	21.71^ab^	72.70^a^	9.96^ab^	33.53^a^	8.26^b^	11.73^a^	25.98	52.38^ab^	14.42
Plant 5 (P)	50.38	8.06	31.50	32.66^ab^	20.92^b^	64.61^c^	9.12^c^	27.85^b^	11.63^a^	11.86^a^	25.61	50.79^b^	15.23
SEM	0.740	1.005	4.010	0.756	0.212	1.699	0.203	1.163	0.745	0.222	0.516	1.058	3.594
p-value	0.046	0.583	0.721	<0.001	<0.001	<0.001	<0.001	0.002	<0.001	<0.001	0.355	0.018	0.531
	------------------------------------------------------------------------------------------ Meal vs Pellet -------------------------------------------------------------------------------------------------------------------												
Contrast p-value	0.788	0.442	0.573	<0.001	0.111	0.005	<0.001	0.011	0.002	<0.001	0.395	0.374	0.303
	--------------------------------------------------------------------------------------- CH processing plants ----------------------------------------------------------------------------------------------------------												
Plant A (M)	49.40	8.87^ab^	35.00^ab^	28.54^b^	20.73	72.53^a^	8.93^ab^	31.18^a^	7.83^c^	11.83	25.63	51.61	15.35
Plant B (M)	49.25	8.88^ab^	34.43^b^	30.63^ab^	21.46	70.34^a^	9.76^a^	31.86^a^	9.11^bc^	11.72	25.63	51.85	15.97
Plant C (M)	48.60	8.76^b^	36.27^ab^	29.06^ab^	20.05	69.15^ab^	8.17^b^	28.19^b^	9.01^c^	11.87	24.22	49.87	15.34
Plant D (M)	48.91	10.21^ab^	43.34^ab^	31.65^a^	20.51	64.85^bc^	8.62^b^	27.28^b^	11.14^ab^	11.87	23.57	48.17	14.99
Plant E (M)	50.02	10.91^a^	43.65^a^	31.62^a^	20.25	64.07^c^	8.77^ab^	27.74^b^	11.37^a^	11.48	25.08	50.12	16.53
SEM	0.615	0.722	2.990	0.738	0.373	1.133	0.276	0.751	0.530	0.285	0.659	1.305	3.723
p-value	0.143	0.019	0.009	0.012	0.062	<0.001	0.010	<0.001	<0.001	0.672	0.114	0.253	0.609
	--------------------------------------------------------------------------------------------------- Overall ----------------------------------------------------------------------------------------------------------------------												
CA Plants	49.48	8.44	33.09	31.74	20.86	65.83	10.07	31.80	10.88	10.80	25.81	52.24	14.34
CH Plants	49.41	9.56	38.74	30.62	20.56	67.91	8.81	29.07	9.80	11.75	24.76	50.20	14.69
SEM	0.562	0.688	2.540	0.633	0.302	1.226	0.228	0.758	0.542	0.190	0.441	0.819	3.212
p-value	0.840	0.098	0.017	0.075	0.408	0.162	<0.001	0.005	0.103	<0.001	0.051	0.044	0.562

For each plant, sample size n = 5.

CHO, total carbohydrate; DM, dry matter; NFC, non-fiber carbohydrate; NDF, neutral detergent fiber; ADF, acid detergent fiber; ADL, acid detergent lignin; HEM, Hemicellulose; NSC, non-structural carbohydrate; M, meal; P, pellet; CA, Canada; CH, China; SEM, standard error of the mean.

a–d Means within a column without a common superscript letter differ (p<0.05).

**Table 3:** Chemical composition profile of canola seeds from different oil processing plants: comparison among bio-oil processing plants and between Canada and China

Items	Basic chemical profile				Protein profile						
	
	DM (%)	Ash (% DM)	EE (% DM)	FA (% DM)	CP (% DM)	SCP (% DM)	SCP (% CP)	NDICP (% DM)	NDICP (% CP)	ADICP (% DM)	ADICP (% CP)
	-------------------------------------------------------------------------------------------------- CA processing plants ---------------------------------------------------------------------------------------------------										
Plant 1	93.67^ab^	3.92^a^	42.29	41.29	23.05	10.42^bc^	45.18^b^	2.67^a^	11.60^a^	1.18^a^	5.14^a^
Plant 2	94.83^a^	3.69^b^	40.66	39.66	22.09	9.43^c^	42.84^b^	2.60^ab^	11.75^a^	1.11^a^	5.03^a^
Plant 3	93.38^bc^	3.97^a^	44.79	43.79	22.81	10.28^bc^	45.21^b^	2.37^b^	10.34^a^	0.97^b^	4.25^b^
Plant 4	91.71^d^	3.80^ab^	43.42	42.42	22.14	11.70^ab^	52.88^a^	2.31^b^	10.44^a^	1.20^a^	5.42^a^
Plant 5	92.22^cd^	3.80^ab^	43.42	42.42	22.13	12.26^a^	55.57^a^	1.96^c^	8.84^b^	1.13^a^	5.12^a^
SEM	0.367	0.053	1.445	1.445	0.267	0.486	2.042	0.073	0.346	0.026	0.137
p-value	<0.001	0.009	0.196	0.196	0.037	<0.001	<0.001	<0.001	<0.001	<0.001	<0.001
	--------------------------------------------------------------------------------------------- CH processing plants --------------------------------------------------------------------------------------------------------										
Plant A	92.31^ab^	3.77	43.09	42.09	22.48^a^	12.49	55.53	2.05	9.15	1.06	4.69
Plant B	92.21^bc^	3.72	46.06	45.06	21.70^b^	12.24	56.39	2.02	9.34	1.18	5.41
Plant C	92.46^ab^	3.87	43.07	42.07	22.40^a^	12.07	54.04	2.00	8.94	1.11	4.97
Plant D	92.79^a^	3.81	43.33	42.33	22.28^a^	11.35	50.89	1.99	8.92	1.08	4.88
Plant E	92.71^c^	3.83	44.37	43.37	22.18^ab^	12.06	54.44	2.06	9.28	1.07	4.82
SEM	0.236	0.043	1.636	1.636	0.168	0.851	3.943	0.084	0.404	0.070	0.314
p-value	<0.001	0.128	0.348	0.348	0.008	0.762	0.676	0.954	0.897	0.607	0.382
	---------------------------------------------------------------------------------------------------- Overall -------------------------------------------------------------------------------------------------------------------------										
CA Plants	93.10	3.84	42.71	41.71	22.46	10.81	48.21	2.39	10.63	1.13	5.06
CH Plants	92.28	3.81	43.91	42.91	22.20	12.04	54.30	2.02	9.11	1.10	4.96
SEM	0.250	0.026	0.848	0.848	0.129	0.461	2.215	0.049	0.215	0.030	0.157
p-value	0.008	0.387	0.191	0.191	0.100	0.003	0.002	<0.001	<0.001	0.338	0.537

For each plant, sample size n = 5.

DM, dry matter; EE, ether extract (crude fat); FA, fatty acid; CP, crude protein; SCP, soluble crude protein; NDICP, neutral detergent-insoluble crude protein; ADICP, acid detergent-insoluble crude protein; CA, Canada; CH, China; SEM, standard error of the mean.

a–d Means within a column without a common superscript letter differ (p<0.05).

**Table 4:** Chemical composition profile of canola seeds from different oil processing plants: comparison among bio-oil processing plants and between Canada and China

Items	Carbohydrate profile												

	CHO (% DM)	Sugar (% DM)	Sugar (% NFC)	NDF (% DM)	ADF (% DM)	ADF (% NDF)	ADL (% DM)	ADL (% NDF)	HEM (% DM)	Cell (% DM)	NFC (% DM)	NFC (% CHO)	NSC (% DM)
	--------------------------------------------------------------------------------------------------- CA processing plants -------------------------------------------------------------------------------------------------------												
Plant 1	30.74	4.95	30.51	17.04	12.18	71.55	5.32^bc^	31.25^ab^	4.85	6.86	16.37	53.06	9.24
Plant 2	33.61	4.85	26.54	17.44	12.45	71.30	5.53^bc^	31.12^ab^	5.05	6.97	18.63	54.91	10.80
Plant 3	28.48	5.74	39.20	16.27	12.03	73.69	4.94^c^	29.81^b^	4.29	7.15	14.44	51.02	9.04
Plant 4	30.65	5.29	35.99	17.52	14.41	76.68	6.44^a^	36.84^a^	4.11	6.96	15.44	50.05	10.07
Plant 5	30.71	5.74	34.42	15.94	12.31	77.24	5.89^ab^	36.60^a^	3.69	6.47	16.59	54.55	9.89
SEM	1.462	0.459	3.760	0.521	0.349	2.499	0.259	1.592	0.514	0.226	1.532	2.491	3.062
p-value	0.122	0.490	0.165	0.147	0.048	0.285	0.001	0.009	0.336	0.282	0.227	0.364	0.824
	--------------------------------------------------------------------------------------------------- CH processing plants -------------------------------------------------------------------------------------------------------												
Plant A	30.68	6.90	39.02	15.27	12.05	78.74	5.27	34.45	3.31	6.71	17.51	56.58	11.42
Plant B	28.50	5.69	38.90	15.59	12.17	78.86	5.85	37.53	3.36	6.26	14.89	51.99	10.03
Plant C	30.66	6.77	42.58	15.58	11.54	74.79	5.91	37.90	4.04	5.63	17.08	55.05	13.02
Plant D	30.57	5.56	34.73	15.91	11.44	72.59	5.64	35.67	4.47	5.80	16.65	54.22	11.22
Plant E	29.62	5.15	35.41	16.52	11.81	71.63	5.61	34.03	4.71	6.20	15.16	50.80	9.66
SEM	1.613	0.790	5.884	0.814	0.345	3.470	0.262	1.326	0.705	0.412	1.763	3.299	3.217
p-value	0.585	0.368	0.721	0.477	0.468	0.062	0.246	0.111	0.064	0.328	0.387	0.300	0.140
	----------------------------------------------------------------------------------------------------- Overall ----------------------------------------------------------------------------------------------------------------------------												
CA Plants	30.92	5.30	32.60	16.96	12.49	73.98	5.65	33.20	4.45	6.88	16.52	52.75	10.04
CH Plants	30.07	5.99	38.46	15.87	11.77	74.52	5.67	35.88	4.14	6.09	16.26	53.59	10.38
SEM	0.815	0.275	2.975	0.439	0.173	1.660	0.201	0.788	0.364	0.168	1.071	2.173	2.793
p-value	0.361	0.076	0.032	0.004	0.003	0.762	0.920	0.017	0.387	<0.001	0.769	0.554	0.681

For each plant, sample size n = 5.

CHO, total carbohydrate; DM, dry matter; NFC, non-fiber carbohydrate; NDF, neutral detergent fiber; ADF, acid detergent fiber; ADL, acid detergent lignin; HEM, Hemicellulose; Cell, cellulose, calculated as ADF-ADL; CA, Canada; CH, China; SEM, standard error of the mean.

a–c Means within a column without a common superscript letter differ (p<0.05).

**Table 5:** Energy profile of co-products from different oil processing plants (canola meals and pellets): comparison among bio-oil processing plants and between Canada and China

Items	Digestible nutrients profile (% DM)				

	tdNDF	tdNFC	tdCP	tdFA	TDN_1x_
	-------------------------- CA processing plants ---------------------------				
Plant 1 (M)	4.34^c^	25.44	45.62^a^	0.56	65.67^b^
Plant 2 (M)	4.38^c^	25.13	39.98^b^	0.55	63.75^b^
Plant 3 (P)	5.04^ab^	26.45	40.84^ab^	1.21	68.07^a^
Plant 4 (P)	4.52^bc^	25.46	40.74^b^	0.83	65.59^b^
Plant 5 (P)	5.63^a^	25.09	40.94^ab^	0.38	65.54^b^
SEM	0.331	0.506	0.223	0.576	0.783
p-value	<0.001	0.356	0.001	0.574	0.001
	------------------------------- Meal vs Pellet -------------------------------				
Contrast p-value	<0.001	0.398	0.846	0.458	0.002
	----------------------------- CH processing plants ------------------------				
Plant A (M)	5.48^ab^	25.12	41.85^bc^	0.38	66.22
Plant B (M)	4.63^b^	25.11	42.16^abc^	0.02	65.00
Plant C (M)	6.29^a^	23.74	42.43^ab^	0.35	66.24
Plant D (M)	6.43^a^	23.10	43.03^a^	0.10	65.79
Plant E (M)	6.14^a^	24.60	41.20^c^	0.03	65.01
SEM	0.295	0.644	0.317	0.210	0.521
p-value	<0.001	0.111	0.002	0.599	0.182
	----------------------------- CH processing plants --------------------------				
CA Plants	4.64	25.65	40.89	0.67	65.62
CH Plants	5.86	24.26	42.13	0.20	65.67
SEM	0.244	0.374	0.250	0.299	0.493
p-value	<0.001	0.006	<0.001	0.055	0.926

For each plant, sample size n = 5.

DM, dry matter; tdNDF, total digestible neutral detergent fiber; tdNFC, total digestible non-fiber carbohydrate; tdCP, total digestible crude protein; tdFA, total digestible fatty acids; TDN_1x_, total digestible nutrients at one time maintenance level; M, meal; P, pellet; CA, Canada; CH, China; SEM, standard error of the mean.

a–c Means within a column without a common superscript letter differ (p<0.05).

**Table 6:** Energy profile of co-products from different oil processing plants (canola meals and pellets): comparison among bio-oil processing plants and between Canada and China

Items	Energy values (Mcal/kg DM)			

	ME_3x_	NE_Lp3x_	NE_m3x_	NE_g3x_
	---------------------------- CA processing plants --------------------------			
Plant 1 (M)	2.73^b^	1.75^ab^	1.81^ab^	1.18^ab^
Plant 2 (M)	2.65^c^	1.70^c^	1.73^c^	1.12^c^
Plant 3 (P)	2.81^a^	1.79^a^	1.87^a^	1.23^a^
Plant 4 (P)	2.72^b^	1.74^bc^	1.80^b^	1.17^bc^
Plant 5 (P)	2.72^b^	1.74^bc^	1.80^bc^	1.17^bc^
SEM	0.026	0.015	0.022	0.020
p-value	<0.001	<0.001	<0.001	<0.001
	------------------------------- Meal vs Pellet ----------------------------------			
Contrast p-value	<0.001	0.003	0.001	0.002
	------------------------- CH processing plants ----------------------------			
Plant A (M)	2.76	1.76	1.83	1.20
Plant B (M)	2.72	1.75	1.80	1.17
Plant C (M)	2.77	1.77	1.83	1.20
Plant D (M)	2.75	1.77	1.83	1.20
Plant E (M)	2.71	1.74	1.78	1.16
SEM	0.020	0.011	0.017	0.015
p-value	0.086	0.066	0.071	0.106
	------------------------------------- Overall --------------------------------------			
CA Plants	2.73	1.75	1.80	1.17
CH Plants	2.74	1.76	1.82	1.19
SEM	0.019	0.010	0.016	0.014
p-value	0.382	0.320	0.397	0.347

For each plant, sample size n = 5.

DM, dry matter; ME_3x_, metabolizable energy for gain at three times the maintenance level; NE_Lp3x_, net energy for lactation at a productive level of intake three times the maintenance level; NE_m3x_, net energy for maintenance; NE_g3x_, net energy for gain; M, meal; P, pellet; CA, Canada; CH, China; SEM, standard error of the mean.

a–c Means within a column without a common superscript letter differ (p<0.05).

**Table 7:** Energy profile of canola seeds from different oil processing plants: comparison among bio-oil processing plants and between Canada (CA) and China (CH)

Items	Digestible nutrients profile (%DM)				

	tdNDF	tdNFC	tdCP	tdFA	TDN_1x_
	---------------------------------- CA processing plants ----------------------------------				
Plant 1	3.29	16.05	22.58	41.29	127.82
Plant 2	3.47	18.26	21.66	39.66	125.55
Plant 3	3.44	14.15	22.43	43.79	131.49
Plant 4	2.89	15.12	21.66	42.42	128.11
Plant 5	2.75	16.25	21.69	42.42	129.06
SEM	0.246	1.502	0.268	1.445	1.743
p-value	0.157	0.226	0.038	0.196	0.136
	---------------------------------- CH processing plants -------------------------------				
Plant A	2.75	17.16	22.06^a^	42.09	129.61
Plant B	2.52	14.60	21.23^b^	45.06	132.76
Plant C	2.45	16.74	21.95^a^	42.07	128.81
Plant D	2.84	16.31	21.85^a^	42.33	129.24
Plant E	3.11	14.86	21.75^ab^	43.37	130.30
SEM	0.279	1.728	0.180	1.636	2.098
p-value	0.145	0.387	0.006	0.348	0.383
	------------------------------------------ Overall ------------------------------------------				
CA Plants	3.15	16.18	22.01	41.71	129.75
CH Plants	2.77	15.93	21.76	42.91	128.07
SEM	0.146	1.049	0.135	0.848	1.275
p-value	0.023	0.770	0.126	0.191	0.328

For each plant, sample size n = 5.

DM, dry matter; tdNDF, total digestible neutral detergent fiber; tdNFC, total digestible non-fiber carbohydrate; tdCP, total digestible crude protein; tdFA, total digestible fatty acids; TDN_1x_, total digestible nutrients at one time maintenance level; M, meal; P, pellet; CA, Canada; CH, China; SEM, standard error of the mean.

a,b Means within a column without a common superscript letter differ (p<0.05).

**Table 8:** Energy profile of canola seeds from different oil processing plants: comparison among bio-oil processing plants and between Canada and China

Items	Energy values (Mcal/kg DM)			

	ME_3x_	NE_Lp3x_	NE_m3x_	NE_g3x_
	----------------------------- CA processing plants ---------------------------------			
Plant 1	4.64	3.08	3.31	2.41
Plant 2	4.55	3.02	3.25	2.36
Plant 3	4.76	3.18	3.41	2.48
Plant 4	4.64	3.09	3.32	2.41
Plant 5	4.67	3.11	3.34	2.43
SEM	0.060	0.048	0.046	0.036
p-value	0.122	0.149	0.132	0.129
	----------------------------- CH processing plants -----------------------------			
Plant A	4.70	3.12	3.36	2.45
Plant B	4.79	3.21	3.44	2.50
Plant C	4.67	3.11	3.33	2.43
Plant D	4.68	3.12	3.34	2.44
Plant E	4.71	3.15	3.37	2.46
SEM	0.072	0.057	0.055	0.043
p-value	0.461	0.426	0.390	0.454
	---------------------------------- Overall -----------------------------------------------			
CA Plants	4.65	3.09	3.32	2.42
CH Plants	4.71	3.14	3.36	2.45
SEM	0.034	0.028	0.026	0.020
p-value	0.161	0.162	0.173	0.143

For each plant, sample size n = 5.

DM, dry matter; ME_3x_, metabolizable energy for gain at three times the maintenance level; NE_Lp3x_, net energy for lactation at a productive level of intake three times the maintenance level; NE_m3x_, net energy for maintenance; NE_g3x_, net energy for gain; CA, Canada; CH, China; SEM, standard error of the mean.

**Table 9:** Protein fractions profile of co-products from different oil processing plants (canola meals and pellets): comparison among bio-oil processing plants and between Canada and China

Items	% CP					% TP			% DM			
		
	PA2	PB1	PB2	PC	TP	PA2	PB1	PB2	PA2	PB1	PB2	PC
	---------------------------------------------------------------------------------------- CA processing plants ------------------------------------------------------------------------------ ---------------------------											
Plant 1 (M)	16.63^b^	65.08^ab^	12.17^ab^	5.80^a^	94.20^b^	17.65^b^	69.06^ab^	12.88^ab^	7.08^b^	27.74^a^	5.19^abc^	2.47^a^
Plant 2 (M)	17.19^ab^	61.45^b^	15.44^a^	5.98^a^	94.02^b^	18.29^ab^	65.43^b^	16.40^a^	7.05^ab^	25.11^b^	6.30^a^	2.45^ab^
Plant 3 (P)	19.00^ab^	66.44^a^	9.75^b^	4.87^b^	95.13^a^	19.98^ab^	69.90^a^	10.25^b^	7.92^ab^	27.60^a^	4.06^bc^	2.02^c^
Plant 4 (P)	20.55^a^	64.62^ab^	8.82^b^	5.69^a^	94.31^b^	21.78^a^	68.50^ab^	9.31^b^	8.57^a^	26.95^a^	3.67^c^	2.37^ab^
Plant 5 (P)	17.82^ab^	63.40^ab^	13.35^ab^	5.49^a^	94.51^b^	18.86^ab^	67.15^ab^	14.11^ab^	7.46^ab^	26.47^ab^	5.58^ab^	2.30^b^
SEM	1.070	1.155	1.216	0.225	0.225	1.100	1.327	1.290	0.468	0.517	0.487	0.090
p-value	0.017	0.012	0.003	<0.001	<0.001	0.018	0.029	0.003	0.037	0.003	0.002	<0.001
	------------------------------------------------------------------------------------------ Meal vs Pellet -----------------------------------------------------------------------------------------------------------------											
Contrast p-value	0.008	0.053	0.003	<0.001	<0.001	0.010	0.124	0.003	0.014	0.110	0.002	<0.001
	------------------------------------------------------------------------------------------ CH processing plants ------------------------------------------------------------------------------------------------------											
Plant A (M)	22.32^b^	66.99^a^	6.01^b^	5.00^bc^	95.00^ab^	23.52^bc^	70.49^a^	6.34^b^	9.53^b^	28.52	2.58^b^	2.14^bc^
Plant B (M)	21.77^bc^	62.16^b^	9.66^a^	6.60^a^	93.40^c^	23.31^bc^	66.55^ab^	10.34^a^	9.45^b^	26.86	4.18^a^	2.85^a^
Plant C (M)	25.46^a^	63.71^ab^	6.08^b^	4.75^c^	95.25^a^	26.73^a^	66.89^ab^	6.39^b^	11.01^a^	27.56	2.63^b^	2.05^c^
Plant D (M)	23.24^ab^	62.41^b^	9.61^a^	4.74^c^	95.26^a^	24.40^ab^	65.52^b^	10.08^a^	10.19^ab^	27.36	4.23^a^	2.08^c^
Plant E (M)	19.36^c^	65.01^ab^	9.88^a^	5.74^b^	94.26^b^	20.54^c^	68.97^ab^	10.49^a^	8.16^c^	27.41	4.17^a^	2.42^b^
SEM	0.862	1.052	0.978	0.209	0.209	0.915	1.049	1.035	0.341	0.486	0.438	0.091
p-value	<0.001	0.021	<0.001	<0.001	<0.001	<0.001	0.016	<0.001	<0.001	0.183	<0.001	<0.001
	----------------------------------------------------------------------------------------------------- Overall ---------------------------------------------------------------------------------------------------------------------											
CA Plants	17.11	63.48	13.48	5.86	94.14	18.18	67.44	14.32	7.15	26.22	5.62	2.45
CH Plants	22.51	64.01	8.18	5.33	94.67	23.77	67.61	8.65	9.71	27.54	3.53	2.29
SEM	0.851	0.939	1.023	0.245	0.245	0.875	0.988	1.089	0.365	0.443	0.435	0.104
p-value	<0.001	0.636	<0.001	0.075	0.075	<0.001	0.887	<0.001	<0.001	0.082	<0.001	0.192

For each plant, sample size n = 5.

CP, crude protein; TP, true protein; DM, dry matter; PA2, soluble true protein; PB1, moderately degradable protein; PB2, slowly degradable protein; PC, unavailable crude protein; M, meal; P, pellet; CA, Canada; CH, China; SEM, standard error of the mean.

a–c Means within a column without a common superscript letter differ (p<0.05).

**Table 10:** Protein fractions profile of canola seeds from different oil processing plants: comparison among bio-oil processing plants and between Canada and China

Items	% CP					% TP			% DM			
		
	PA2	PB1	PB2	PC	TP	PA2	PB1	PB2	PA2	PB1	PB2	PC
	------------------------------------------------------------------------------------------------- CA processing plants -----------------------------------------------------------------------------------------------------------											
Plant 1	53.06	35.32	6.46^a^	5.14^a^	94.86^b^	55.93	37.22	6.81^a^	12.24	8.15	1.49^a^	1.18^a^
Plant 2	54.91	33.81	6.72^a^	5.03^a^	94.97^b^	57.84	35.66	7.08^a^	12.01	7.38	1.49^a^	1.11^a^
Plant 3	51.02	39.11	6.10^ab^	4.25^b^	95.75^a^	53.30	40.90	6.37^ab^	11.50	8.85	1.39^ab^	0.97^b^
Plant 4	50.05	39.49	5.02^b^	5.42^a^	94.58^b^	52.91	41.74	5.31^b^	11.09	8.74	1.11^b^	1.20^a^
Plant 5	54.55	37.09	3.72^c^	5.12^a^	94.89^b^	57.50	39.16	3.92^c^	11.94	8.12	0.83^c^	1.13^a^
SEM	2.410	2.870	0.308	0.137	0.137	2.619	2.997	0.325	0.741	0.779	0.068	0.026
p-value	0.364	0.187	<0.001	<0.001	<0.001	0.341	0.194	<0.001	0.404	0.1994	<0.001	<0.001
	------------------------------------------------------------------------------------------------- CH processing plants ------------------------------------------------------------------------------------------------------											
Plant A	56.58	34.23	4.34	4.69	95.31	59.33	35.92	4.56	12.72	7.71	0.97	1.06
Plant B	51.99	38.75	3.87	5.41	94.59	54.95	40.96	4.09	11.30	8.40	0.83	1.18
Plant C	55.05	36.02	3.97	4.97	95.03	57.89	37.94	4.17	12.35	8.04	0.89	1.11
Plant D	54.22	36.86	4.04	4.88	95.12	56.95	38.8	4.25	12.09	8.21	0.90	1.08
Plant E	50.80	39.93	4.46	4.82	95.18	53.36	41.96	4.68	11.27	8.86	0.99	1.07
SEM	3.298	3.199	0.477	0.314	0.314	3.341	3.447	0.493	0.784	0.700	0.109	0.066
p-value	0.300	0.280	0.860	0.382	0.382	0.302	0.287	0.866	0.186	0.402	0.807	0.607
	---------------------------------------------------------------------------------------------------- Overall -----------------------------------------------------------------------------------------------------------------------------											
CA Plants	52.75	36.54	5.62	5.05	94.95	55.59	38.52	5.91	11.87	1.26	8.21	1.13
CH Plants	53.59	37.29	4.14	4.96	95.04	56.37	39.27	4.35	11.92	0.92	8.27	1.10
SEM	2.173	2.138	0.229	0.157	0.157	2.224	2.291	0.239	0.543	0.052	0.445	0.030
p-value	0.554	0.588	<0.001	0.537	0.537	0.597	0.608	<0.001	0.874	<0.001	0.829	0.338

For each plant, sample size n = 5.

CP, crude protein; TP, true protein; DM, dry matter; PA2, soluble true protein; PB1, moderately degradable protein; PB2, slowly degradable protein; PC, unavailable crude protein; CA, Canada; CH, China; SEM, standard error of the mean.

a–c Means within a column without a common superscript letter differ (p<0.05).

**Table 11:** Carbohydrate fractions profile of co-products from different oil processing plants (canola meals and pellets): comparison among bio-oil processing plants and between Canada and China

Items	CHO	CA4	CB1	CB2	CB3	CC	CA4	CB2	CB3	CC
		
	% DM	% CHO					% DM			
	--------------------------------------------------------------------------------------- CA processing plants ------------------------------------------------------------------------------------------------------------									
Plant 1 (M)	48.98	17.95	2.04	33.17	23.92^ab^	23.18^bc^	8.72	16.27	11.76^b^	11.32^b^
Plant 2 (M)	50.14	15.69	1.99	32.94	24.50^ab^	25.43^a^	7.97	16.49	12.33^b^	12.75^a^
Plant 3 (P)	48.78	18.63	2.05	34.11	21.36^b^	19.00^d^	9.10	16.71	10.45^b^	9.25^c^
Plant 4 (P)	49.81	19.45	2.01	30.92	21.09^b^	23.89^ab^	9.58	15.43	10.55^b^	11.89^ab^
Plant 5 (P)	50.38	15.81	1.99	32.57	28.52^a^	21.88^c^	8.06	16.36	14.41^a^	11.02^b^
SEM	0.740	2.613	0.032	2.092	2.000	0.488	1.005	1.047	0.625	0.296
p-value	0.046	0.609	0.073	0.785	0.002	<0.001	0.583	0.909	<0.001	<0.001
	-------------------------------------------------------------------------------------------- Meal vs Pellet -------------------------------------------------------------------------------------------------------------------									
Contrast p-value	0.788	0.476	0.913	0.757	0.529	<0.001	0.442	0.812	0.531	<0.001
	----------------------------------------------------------------------------------------- CH processing plants -----------------------------------------------------------------------------------------------------------									
Plant A (M)	49.40	18.06	2.03	31.75^ab^	22.19^b^	21.44^ab^	8.87^ab^	15.67^a^	10.82^b^	10.59^ab^
Plant B (M)	49.25	18.11	2.03	32.43^a^	24.24^ab^	23.42^a^	8.88^ab^	15.93^a^	11.95^ab^	11.52^a^
Plant C (M)	48.60	18.07	2.06	29.75^ab^	24.91^ab^	19.62^b^	8.76^b^	14.46^ab^	12.10^ab^	9.54^b^
Plant D (M)	48.91	20.91	2.04	25.21^b^	30.32^a^	20.69^b^	10.21^ab^	12.36^b^	14.80^a^	10.12^b^
Plant E (M)	50.02	21.83	2.00	26.29^ab^	27.68^ab^	21.06^ab^	10.91^a^	13.17^ab^	13.82^ab^	10.52^ab^
SEM	0.615	1.551	0.025	1.692	1.714	0.662	0.722	0.894	0.764	0.382
p-value	0.143	0.019	0.153	0.012	0.013	0.010	0.019	0.014	0.007	0.008
	-------------------------------------------------------------------------------------------- Overall ------------------------------------------------------------------------------------------------------------------------------									
CA Plants	49.48	17.25	2.02	32.93	24.04	24.17	8.44	16.30	11.91	11.96
CH Plants	49.41	19.42	2.02	28.81	26.02	21.15	9.56	14.21	12.81	10.44
SEM	0.562	1.279	0.023	1.403	1.275	0.547	0.688	0.772	0.606	0.320
p-value	0.840	0.040	0.906	0.010	0.200	<0.001	0.098	0.009	0.214	<0.001

For each plant, sample size n = 5.

CHO, carbohydrates; CA4, water soluble carbohydrate; CB1, rapidly degradable CHO fraction; CB2, soluble fiber; CB3, digestible fiber; CC, indigestible fiber; DM, dry matter; M, meal; P, pellet; CA, Canada; CH, China; SEM, standard error of the mean.

a–d Means within a column without a common superscript letter differ (p<0.05).

**Table 12:** Carbohydrate fractions profile of canola seeds from different oil processing plants: comparison among bio-oil processing plants and between Canada and China

Items	% DM	% CHO				% DM			
		
	CHO	CA4	CB2	CB3	CC	CA4	CB2	CB3	CC
	---------------------------------------------------------------------------------------------- CA processing plants ---------------------------------------------------------------------------------------------------								
Plant 1	30.74	16.14	28.83^bc^	30.04	12.76^bc^	4.95	8.80^ab^	9.21	3.91^bc^
Plant 2	33.61	14.36	29.29^bc^	28.58	13.26^bc^	4.85	9.73^ab^	9.42	4.35^ab^
Plant 3	28.48	20.02	25.99^c^	31.49	11.84^c^	5.74	7.30^b^	8.99	3.33^c^
Plant 4	30.65	17.63	35.04^ab^	29.55	15.46^a^	5.29	10.75^a^	8.98	4.75^a^
Plant 5	30.71	18.71	37.66^a^	25.40	14.14^ab^	5.74	11.51^a^	7.87	4.30^ab^
SEM	1.462	1.578	2.150	2.218	0.622	0.459	0.692	0.496	0.224
p-value	0.122	0.141	0.002	0.315	<0.001	0.500	0.003	0.248	0.003
	------------------------------------------------------------------------------------------------- CH processing plants ------------------------------------------------------------------------------------------------------								
Plant A	30.68	22.50	33.59	25.24	12.65	6.90	10.37	7.62	3.88
Plant B	28.50	19.68	36.88	27.96	14.05	5.70	10.45	7.88	3.99
Plant C	30.66	22.47	31.57	24.63	14.18	6.77	9.61	7.38	4.32
Plant D	30.57	18.35	32.54	26.2	13.53	5.56	9.96	7.95	4.15
Plant E	29.62	17.69	36.75	29.63	13.47	5.15	10.90	8.69	4.00
SEM	1.613	2.661	2.912	2.794	0.628	0.790	0.984	0.690	0.261
p-value	0.585	0.457	0.331	0.258	0.245	0.368	0.600	0.254	0.358
	----------------------------------------------------------------------------------------------------- Overall --------------------------------------------------------------------------------------------------------------------------								
CA Plants	30.92	17.07	31.44	28.93	13.56	5.30	9.66	8.90	4.18
CH Plants	30.07	20.17	34.21	26.82	13.60	5.99	10.28	7.98	4.09
SEM	0.815	1.164	1.849	1.78	0.483	0.275	0.531	0.392	0.185
p-value	0.361	0.022	0.077	0.107	0.920	0.076	0.250	0.006	0.595

For each plant, sample size n = 5.

DM, dry matter; CHO, carbohydrates; CA4, water soluble carbohydrate; CB2, soluble fiber; CB3, digestible fiber; CC, indigestible fiber; CA, Canada; CH, China; SEM, standard error of the mean.

a–c Means within a column without a common superscript letter differ (p<0.05).

**Table 13:** Ruminal degradation and intestinal digestion profile of carbohydrate in co-products from different oil processing plants (canola meal and pellet): comparison among bio-oil processing plants and between Canada and China

Items	Rumen degradable profile (% DM)				Rumen undegradable profile (% DM)					Intestinal digestible profile (% DM)			
		
	RDCA4	RDCB2	RDCB3	Total RDC	RUCA4	RUCB2	RUCB3	RUCC	Total RUC	DIGCA4	DIGCB2	DIGCB3	DIGFC
	--------------------------------------------------------------------------------------------- CA processing plants -----------------------------------------------------------------------------------------------------												
Plant 1 (M)	6.71	12.51	5.88^b^	25.90	2.01	3.75	5.88^b^	11.32^b^	23.25^b^	2.01	3.75	5.88^b^	11.90^b^
Plant 2 (M)	6.13	12.69	6.16^b^	25.87	1.84	3.81	6.16^b^	12.75^a^	24.78^a^	1.84	3.81	6.16^b^	12.07^b^
Plant 3 (P)	7.00	12.86	5.22^b^	25.97	2.10	3.86	5.22^b^	9.25^c^	20.66^c^	2.10	3.86	5.22^b^	11.44^b^
Plant 4 (P)	7.37	11.87	5.28^b^	25.31	2.21	3.56	5.28^b^	11.89^ab^	23.22^b^	2.21	3.56	5.28^b^	12.30^b^
Plant 5 (P)	6.20	12.59	7.21^a^	26.89	1.86	3.78	7.21^a^	11.02^b^	24.10^ab^	1.86	3.78	7.21^a^	13.11^a^
SEM	0.773	0.904	0.312	0.492	0.232	0.270	0.312	0.260	0.449	0.232	0.270	0.312	0.314
p-value	0.584	0.909	<0.001	0.109	0.579	0.908	<0.001	<0.001	<0.001	0.579	0.908	<0.001	<0.001
	---------------------------------------------------------------------------------------------- Meal vs Pellet ----------------------------------------------------------------------------------------------------------------												
Contrast p-value	0.443	0.814	0.536	0.600	0.440	0.809	0.536	<0.001	<0.001	0.440	0.809	0.536	0.827
	-------------------------------------------------------------------------------------------- CH processing plants --------------------------------------------------------------------------------------------------------												
Plant A (M)	6.82^ab^	12.05^a^	5.41^b^	25.13	2.05^ab^	3.61^a^	5.41^b^	10.59^ab^	22.00^ab^	2.05^ab^	3.61^a^	5.41^b^	11.33^b^
Plant B (M)	6.83^ab^	12.25^a^	5.97^ab^	25.68	2.05^ab^	3.68^a^	5.97^ab^	11.52^a^	23.47^a^	2.05^ab^	3.68^a^	5.97^ab^	11.89^ab^
Plant C (M)	6.74^b^	11.13^ab^	6.05^ab^	24.68	2.02^b^	3.34^ab^	6.05^ab^	9.54^b^	21.18^b^	2.02^b^	3.34^ab^	6.05^ab^	11.64^ab^
Plant D (M)	7.86^ab^	9.51^b^	7.40^a^	25.53	2.36^ab^	2.85^b^	7.40^a^	10.12^b^	22.97^a^	2.36^ab^	2.85^b^	7.40^a^	12.84^a^
Plant E (M)	8.40^a^	10.13^ab^	6.91^ab^	26.20	2.52^a^	3.04^ab^	6.91^ab^	10.52^ab^	23.23^a^	2.52^a^	3.04^ab^	6.91^ab^	12.70^a^
SEM	0.555	0.688	0.381	0.387	0.166	0.206	0.381	0.382	0.435	0.166	0.206	0.381	0.317
p-value	0.018	0.014	0.007	0.074	0.018	0.014	0.007	0.008	0.004	0.018	0.014	0.007	0.008
	----------------------------------------------------------------------------------------------------- Overall ------------------------------------------------------------------------------------------------------------------												
CA Plants	6.50	12.54	5.95	25.81	1.95	3.76	5.95	11.96	23.86	1.95	3.76	5.95	11.91
CH Plants	7.36	10.94	6.40	25.45	2.21	3.28	6.40	10.44	22.55	2.21	3.28	6.40	12.12
SEM	0.527	0.594	0.303	0.319	0.161	0.178	0.303	0.321	0.394	0.161	0.178	0.303	0.266
p-value	0.097	0.009	0.215	0.344	0.101	0.008	0.215	<0.001	0.009	0.101	0.008	0.215	0.510

For each plant, sample size n = 5.

DM, dry matter; RDCA4, rumen degradable water-soluble carbohydrates; RDCB2, RD soluble fiber; RDCB3, RD digestible fiber; Total RDC, total RD carbohydrates; RUCA4, rumen undegradable water soluble CHO; RUCB2, rumen undegradable soluble fiber; RUCB3, rumen undegradable digestible fiber; Total RUC, total rumen undegradable CHO; RUCC, indigestible fiber; DIGCA4, digestible water-soluble CHO; DIGCB2, digestible soluble fiber; DIGCB3, digestible fiber; DIGFC, digestible feed CHO; M, meal; P, pellet; CA, Canada; CH, China; SEM, standard error of the mean.

a–c Means within a column without a common superscript letter differ (p<0.05).

**Table 14:** Ruminal degradation and intestinal digestion profile of carbohydrate in canola seeds from different oil processing plants: comparison among bio-oil processing plants and between Canada and China

Items	Carbohydrate profile												

	Rumen degradable profile				Rumen undegradable profile					Intestinal digestible profile			
		
	RDCA4 (% DM)	RDCB2 (% DM)	RDCB3 (% DM)	Total RDC (% DM)	RUCA4 (% DM)	RUCB2 (% DM)	RUCB3 (% DM)	RUCC (% DM)	TotalRUC (% DM)	DIGCA4 (% DM)	DIGCB2 (% DM)	DIGCB3 (% DM)	DIGFC (% DM)
	------------------------------------------------------------------------------------------------- CA processing plants ----------------------------------------------------------------------------------------------------------------												
Plant 1	3.80	6.77^ab^	4.61	15.18^ab^	1.14	2.03^ab^	4.61	3.91^bc^	11.71^ab^	1.14	2.03^ab^	4.61	7.77
Plant 2	3.73	7.48^ab^	4.71	15.92^ab^	1.12	2.24^ab^	4.71	4.35^ab^	12.41^ab^	1.12	2.24^ab^	4.71	8.08
Plant 3	4.42	5.61^b^	4.50	14.52^b^	1.33	1.68^b^	4.50	3.53^c^	10.82^b^	1.33	1.68^b^	4.50	7.51
Plant 4	4.07	8.27^a^	4.49	16.83^ab^	1.22	2.48^a^	4.49	4.75^a^	12.96^a^	1.22	2.48^a^	4.49	8.19
Plant 5	4.42	8.86^a^	3.93	17.21^a^	1.33	2.66^a^	3.93	4.30^ab^	12.20^ab^	1.33	2.66^a^	3.93	7.92
SEM	0.354	0.532	0.248	0.586	0.106	0.160	0.248	0.224	0.389	0.106	0.160	0.248	0.307
p-value	0.487	0.003	0.244	0.020	0.481	0.003	0.244	0.003	0.010	0.481	0.003	0.244	0.523
	------------------------------------------------------------------------------------------------- CH processing plants ---------------------------------------------------------------------------------------------------------------												
Plant A	5.31	7.97	3.81	16.98	1.59	2.39	3.81	3.88	11.64	1.59	2.39	3.81	7.75^ab^
Plant B	4.38	8.04	3.94	16.17	1.32	2.41	3.94	3.99	11.57	1.32	2.41	3.94	7.60^ab^
Plant C	5.21	7.39	3.69	16.29	1.56	2.22	3.69	4.32	11.79	1.56	2.22	3.69	7.47^b^
Plant D	4.28	7.66	3.98	15.92	1.28	2.30	3.98	4.15	11.70	1.28	2.30	3.98	7.56^ab^
Plant E	3.96	8.38	4.35	16.69	1.19	2.52	4.35	4.00	12.04	1.19	2.52	4.35	8.05^a^
SEM	0.608	0.757	0.345	1.023	0.182	0.226	0.345	0.261	0.611	0.182	0.226	0.345	0.452
p-value	0.365	0.603	0.256	0.484	0.364	0.595	0.256	0.358	0.519	0.364	0.595	0.256	0.043
	--------------------------------------------------------------------------------------------------------- Overall ---------------------------------------------------------------------------------------------------------------------------												
CA Plants	4.07	7.43	4.45	15.94	1.22	2.23	4.45	4.18	12.15	1.22	2.23	4.45	7.90
CH Plants	4.61	7.91	3.99	16.52	1.38	2.37	3.99	4.09	11.82	1.38	2.37	3.99	7.75
SEM	0.211	0.409	0.196	0.524	0.063	0.122	0.196	0.185	0.379	0.063	0.122	0.196	0.265
p-value	0.076	0.252	0.006	0.218	0.077	0.249	0.006	0.595	0.235	0.077	0.249	0.006	0.419

For each plant, sample size n = 5.

RDCA4, rumen degradable water-soluble carbohydrates; RDCB2, rumen degradable soluble fiber; RDCB3, rumen degradable digestible fiber; Total RDC, total rumen degradable carbohydrates; RUCA4, rumen undegradable water soluble carbohydrates; RUCB2, rumen undegradable soluble fiber; RUCB3, rumen undegradable digestible fiber; TotalRUC, total rumen undegradable carbohydrates; RUCC, indigestible fiber; DIGCA4, digestible water-soluble carbohydrates; DIGCB2, digestible soluble fiber; DIGCB3, digestible fiber; DIGFC, digestible feed carbohydrate; DM, dry matter; CA, Canada; CH, China; SEM, standard error of the mean.

a–c Means within a column without a common superscript letter differ (p<0.05).

**Table 15:** Ruminal degradation and intestinal digestion profile of protein in co-products from different oil processing plants (canola meal and pellet): comparison among bio-oil processing plants and between Canada and China

Items	Rumen degradable profile					Rumen undegradable profile					Intestinal digestible profile		
		
	(% DM)	(% DM)	(% DM)	(% DM)	(% DM)	(% NDF)	(% DM)	(% DM)	(% DM)	(% DM)	(% DM)	(% DM)	(% DM)
		
	RDPA2	RDPB1	RDPB2	RDPEP	Total RDP	RUPA2	RUPB1	RUPB2	RUPC	Total RUP	DIGPB1	DIGPB2	DIGFP
	------------------------------------------------------------------------------------------ CA processing plants ----------------------------------------------------------------------------------------------------												
Plant 1 (M)	5.06^b^	11.10^a^	1.73^abc^	17.92	17.92	2.03b	16.65^a^	3.46^abc^	2.47^a^	24.68^a^	16.65^a^	3.46^abc^	20.18^a^
Plant 2 (M)	5.04^ab^	10.04^b^	2.10^a^	17.27	17.27	2.01^ab^	15.07^b^	4.20^a^	2.45^ab^	23.72^bc^	15.07^b^	4.20^a^	19.27^bc^
Plant 3 (P)	5.66^ab^	11.04^a^	1.35^bc^	18.14	18.14	2.26^ab^	16.56^a^	2.70^bc^	2.02^c^	23.55^c^	16.56^a^	2.70^bc^	19.27^bc^
Plant 4 (P)	6.12^a^	10.78^a^	1.22^c^	18.17	18.17	2.45^a^	16.17^a^	2.45^c^	2.37^ab^	23.51^c^	16.17^a^	2.45^c^	18.69^c^
Plant 5 (P)	5.33^ab^	10.59^ab^	1.86^ab^	17.86	17.86	2.13^ab^	15.88^ab^	3.72^ab^	2.30^b^	24.02^b^	15.88^ab^	3.72^ab^	19.61^ab^
SEM	0.334	0.207	0.162	0.240	0.240	0.134	0.311	0.325	0.091	0.165	0.311	0.325	0.199
p-value	0.038	0.003	0.002	0.074	0.074	0.036	0.003	0.002	<0.001	<0.001	0.003	0.002	<0.001
	-------------------------------------------------------------------------------------------- Meal vs Pellet --------------------------------------------------------------------------------------------------------------------												
Contrast p-value	0.014	0.109	0.002	0.034	0.034	0.014	0.112	0.003	<0.001	<0.001	0.112	0.003	0.001
	-------------------------------------------------------------------------------------------- CH processing plants -------------------------------------------------------------------------------------------------------												
Plant A (M)	6.81^b^	11.44	0.86^b^	19.08^bc^	19.08^bc^	2.72^b^	17.16	1.72^b^	2.14^bc^	23.68^ab^	17.16	1.72^b^	18.81^ab^
Plant B (M)	6.75^b^	10.75	1.39^a^	18.88^c^	18.88^c^	2.70^b^	16.12	2.79^a^	2.85^a^	24.47^a^	16.12	2.79^a^	18.91^ab^
Plant C (M)	7.86^a^	11.02	0.88^b^	19.76^a^	19.76^a^	3.14^a^	16.53	1.75^b^	2.05^c^	23.49^b^	16.53	1.75^b^	18.29^b^
Plant D (M)	7.28^ab^	10.95	1.41^a^	19.63^ab^	19.63^ab^	2.91^ab^	16.42	2.82^a^	2.08^c^	24.23^a^	16.42	2.82^a^	19.24^a^
Plant E (M)	5.83^c^	10.95	1.39^a^	18.19^d^	18.19^d^	2.33^c^	16.45	2.78^a^	2.42^b^	23.98^ab^	16.45	2.78^a^	19.23^a^
SEM	0.244	0.194	0.146	0.125	0.125	0.098	0.292	0.292	0.091	0.269	0.292	0.292	0.329
p-value	<0.001	0.186	<0.001	<0.001	<0.001	<0.001	0.185	<0.001	<0.001	0.006	0.185	<0.001	0.039
	------------------------------------------------------------------------------------------- Overall ---------------------------------------------------------------------------------------------------------------------												
CA Plants	5.11	10.64	1.87	17.64	17.64	2.04	15.96	3.74	2.45	24.23	15.96	3.74	19.73
CH Plants	6.94	11.02	1.18	19.12	19.12	2.77	16.53	2.35	2.29	23.91	16.53	2.35	18.87
SEM	0.261	0.177	0.145	0.210	0.210	0.104	0.266	0.290	0.104	0.233	0.266	0.290	0.252
p-value	<0.001	0.082	<0.001	<0.001	<0.001	<0.001	0.083	<0.001	0.192	0.139	0.083	<0.001	0.002

For each plant, sample size n = 5.

DM, dry matter; RDPA2, rumen degradable soluble true protein; RDPB1, RD moderately degradable protein; RDPB2, RD slowly degradable protein; RDPEP, RD peptides; TotalRDP, total RD protein; RUPA2, rumen undegradable soluble true protein; RUPB1, RU moderately degradable protein; RUPB2, rumen undegradable slowly degradable protein; RUPC, rumen undegradable unavailable crude protein; TotalRUP, total RU unavailable protein; DIGPA2, digestible soluble protein; DIGPB1, moderately degradable protein; DIGPB2, digestible slowly degradable protein; DIGFP, digestible feed protein; M, meal; P, pellet; CA, Canada; CH, China; SEM, standard error of the mean.

a–d Means within a column without a common superscript letter differ (p<0.05).

**Table 16:** Ruminal degradation and intestinal digestion profile of protein in canola seeds from different oil processing plants: comparison among bio-oil processing plants and between Canada and China

Items	Rumen degradable profile					Rumen undegradable profile					Intestinal digestible profile		
		
	RDPA2 (% DM)	RDPB1 (% DM)	RDPB2 (% NFC)	RDPEP (% DM)	Total RDP (% DM)	RUPA2 (% NDF)	RUPB1 (% DM)	RUPB2 (% NDF)	RUPC (% DM)	TotalRUP (% DM)	DIGPB1 (% DM)	DIGPB2 (% CHO)	DIGFP (% DM)
	--------------------------------------------------------------------------------------------------- CA processing plants -----------------------------------------------------------------------------------------------------------												
Plant 1	8.74	3.26	0.49^a^	12.50	12.50	3.50	4.89	0.99^a^	1.18^a^	10.56	4.89	0.99^a^	5.89
Plant 2	8.58	2.95	0.50^a^	12.03	12.03	3.44	4.43	0.99^a^	1.11^a^	9.97	4.43	0.99^a^	5.38
Plant 3	8.22	3.54	0.47^ab^	12.21	12.21	3.29	5.31	0.93^a^	0.97^b^	10.50	5.31	0.93^a^	6.20
Plant 4	7.92	3.50	0.37^b^	11.79	11.79	3.17	5.24	0.74^b^	1.20^a^	10.35	5.24	0.74^b^	5.99
Plant 5	8.53	3.25	0.27^c^	12.05	12.05	3.42	4.88	0.55^c^	1.13^a^	9.98	4.88	0.55^c^	5.38
SEM	8.739	0.293	0.023	0.267	0.267	0.215	0.429	0.046	0.027	0.187	0.429	0.046	0.330
p-value	0.408	0.197	<0.001	0.137	0.137	0.405	0.199	<0.001	<0.001	0.074	0.199	<0.001	0.193
	------------------------------------------------------------------------------------------------------ CH processing plants -----------------------------------------------------------------------------------------------------------												
Plant A	9.08	3.08	0.32	12.50	12.50	3.63	4.62	0.65	1.06	9.99	4.62	0.65	5.31
Plant B	8.07	3.36	0.28	11.71	11.71	3.23	5.04	0.56	1.18	10.00	5.04	0.56	5.60
Plant C	8.82	3.22	0.30	12.34	12.34	3.53	4.83	0.59	1.11	10.06	4.83	0.59	5.42
Plant D	8.64	3.28	0.30	12.22	12.22	3.46	4.93	0.60	1.08	10.06	4.93	0.60	5.52
Plant E	8.05	3.54	0.33	11.92	11.92	3.22	5.31	0.66	1.07	10.26	5.31	0.66	5.97
SEM	0.560	0.276	0.036	0.306	0.306	0.224	0.414	0.073	0.066	0.221	0.414	0.073	0.396
p-value	0.187	0.404	0.830	0.068	0.068	0.190	0.404	0.809	0.607	0.666	0.404	0.809	0.406
	---------------------------------------------------------------------------------------------------- Overall -------------------------------------------------------------------------------------------------------------------------------												
CA Plants	8.48	3.28	0.42	12.18	12.18	3.39	4.92	0.84	1.13	10.31	4.92	0.84	5.77
CH Plants	8.51	3.31	0.31	12.13	12.13	3.41	4.97	0.61	1.10	10.09	4.97	0.61	5.58
SEM	0.389	0.178	0.017	0.196	0.196	0.155	0.267	0.035	0.030	0.128	0.267	0.035	0.254
p-value	0.880	0.832	<0.001	0.740	0.740	0.883	0.827	<0.001	0.338	0.072	0.827	<0.001	0.332

For each plant, sample size n = 5.

RDPA2, rumen degradable soluble true protein; RDPB1, rumen degradable moderately degradable protein; RDPB2, rumen degradable slowly degradable protein; RDPEP, rumen degradable peptides; TotalRDP, total rumen degradable protein; RUPA2, rumen undegradable soluble true protein; RUPB1, rumen undegradable moderately degradable protein; RUPB2, rumen undegradable slowly degradable protein; RUPC, rumen undegradable unavailable crude protein; TotalRUP, total rumen undegradable unavailable protein; DIGPA2, digestible soluble protein; DIGPB1, moderately degradable protein; DIGPB2, digestible slowly degradable protein; DIGFP, digestible feed protein; DM, dry matter; NFC, non-fiber carbohydrate; NDF, neutral detergent fiber; CHO, carbohydrates; CA, Canada; CH, China; SEM, standard error of the mean.

a–c Means within a column without a common superscript letter differ (p<0.05).
